# Variation of Microorganisms and Water Quality, and Their Impacts on the Production of *Penaeus vannamei* in Small-Scale Greenhouse Ponds

**DOI:** 10.3390/microorganisms13030546

**Published:** 2025-02-27

**Authors:** Siyu Wu, Haochang Su, Lei Su, Yucheng Cao, Guoliang Wen, Yu Xu, Bin Shen, Shanshan Wu, Yuting Su, Xiaojuan Hu

**Affiliations:** 1 South China Sea Fisheries Research Institute, Chinese Academy of Fishery Sciences, Southern Marine Science and Engineering Guangdong Laboratory (Zhuhai), Guangzhou 510300, China; wusisi98@163.com (S.W.); su.haochang@163.com (H.S.); cyc_169@163.com (Y.C.); gwowen66@163.com (G.W.); xuyublq@163.com (Y.X.); 2 National Engineering Research Center for Marine Aquaculture, Zhejiang Ocean University, Zhoushan 316022, China; shenbin@zjou.edu.cn; 3 Key Laboratory of South China Sea Fishery Resources Exploitation & Utilization, Ministry of Agriculture and Rural Affairs, Guangzhou 510300, China; 4 Guangdong Provincial Key Laboratory of Fishery Ecology and Environment, Guangzhou 510300, China; 5 Guangzhou Resource Environmental Protection Technology Co., Ltd., Guangzhou 510699, China; sul@rcnzy.com (L.S.); wuss@rcnzy.com (S.W.); suyt@rcnzy.com (Y.S.)

**Keywords:** small-scale greenhouse pond, microbial community, water quality, shrimp production, *Penaeus vannamei*

## Abstract

To study the factors affecting *Penaeus vannamei* production in small-scale greenhouse ponds, four ponds in Jiangmen, Guangdong Province, China were selected. This study investigated the variation in the characteristics of bacterial communities and pathogens in pond water and shrimp intestines, as well as water quality factors during the culture stage. Multivariate linear regression equations were used to analyse the potential factors affecting production. The nitrite concentration reached its peak in the mid-culture stage, with a maximum of 16.3 mg·L^−1^, whereas total nitrogen and salinity were highest in the late culture stage, reaching 48.4 mg·L^−1^ and 26, respectively. The dominant bacteria in the pond water were *Marivita* and Rhodobacteraceae, whereas in the shrimp intestines, they were *Bacillus* and *Candidatus Bacilloplasma.* The nitrifying bacteria in the pond water were dominated by *Nitrosomonas* and *Nitrobacter*. Pathogens detected in the pond water included acute hepatopancreatic necrosis disease (AHPND), *Enterocytozoon hepatopenaei* (EHP), and white spot syndrome virus (WSSV). The counts of EHP and the relative abundance of *Ardenticatenales_norank* and *Marivita* in the pond were the main factors affecting the shrimp production (*p* < 0.01). This study indicates that establishing optimal bacterial communities, such as *Marivita*, *Nitrobacter*, and Rhodobacteraceae, and controlling the counts of EHP and AHPND pathogens is crucial for regulating the pond environment and enhancing production.

## 1. Introduction

*Penaeus vannamei* (*P. vannamei*) is the most extensively cultured shrimp species globally, and is known for its strong environmental adaptability, low nutritional feed requirements, and suitability for high-density culture [1]. The *P. vannamei* aquaculture industry has developed rapidly in China, with a variety of culture models including aboveground pond cultures, earthen pond cultures, small greenhouse cultures, integrated multitrophic cultures, and industrialised cultures [2]. The small-scale greenhouse pond for *P. vannamei* belongs to the small greenhouse culture model, which supports two to three crops per year. This model is characterised by ease of management, minimal impact from weather, low cost, and high efficiency [3]. Therefore, it has developed rapidly in recent years and received important attention from the industry.

Microorganisms play important roles as decomposers in shrimp ecosystems. They can decompose the metabolic products in ponds, effectively promoting material cycling in aquaculture ecosystems. At the same time, the stability of the microbial community is an important indicator of the pond environment [4]. Proteobacteria, Firmicutes, and Bacteroidota are common phyla in aquaculture environments [5,6]. Numerous bacteria play a role in degrading organic matter and enhancing water quality, such as the addition of *Bacillus*, which helps decompose organic debris and reduce nitrogenous waste, improving the pond environment [7]. *Nitrosomonas* and *Nitrobacter*, among other nitrifying bacteria, are important for the conversion of inorganic nitrogen, such as nitrite into pond water [8]. The intestinal microbiota of shrimps indicates their health status. Studies have shown that Rhodobacteraceae are the dominant group in the intestinal microbiota of *P. vannamei* and have a higher relative abundance in the intestines of healthy shrimp, making them a key indicator of shrimp health [9]. However, *Candidatus Bacilloplasma* is a pathogenic bacterium that is more frequently found and has a higher relative abundance in the intestines of diseased shrimp than in healthy shrimp, leading to disorders in the bacterial community within the shrimp [10,11]. Therefore, the development of a robust bacterial community is crucial for the growth of healthy shrimp.

In shrimp culture, the focus is on maintaining water quality, which is of paramount importance for ensuring a successful culture and enhancing the economic benefits. The literature reports that poor water quality can cause stress in aquatic animals, making them more susceptible to disease, and even death [12]. The buildup of detrimental nitrogenous substances, including ammonia and nitrite, may negatively affect shrimp health. Optimising the water environment is one of the key factors in improving the efficiency and productivity of cultures, as it promotes the healthy growth of cultured organisms [13]. Therefore, the development of an excellent pond environment is crucial for the growth of healthy shrimp.

Over the past few years, the frequent occurrence of shrimp disease has severely affected the green and high-quality development of the shrimp industry in China. Pathogenic infections are one of the main causes of shrimp diseases. Acute hepatopancreatic necrosis disease (AHPND) [14], *Enterocytozoon hepatopenaei* (EHP) [15], decapod iridescent virus 1 (DIV1) [16], and white spot syndrome virus (WSSV) [17] are common pathogens that currently affect shrimp cultures. The regular monitoring of the prevalence of these common pathogens during culture is important for ensuring healthy shrimp growth.

In the South China region, small-scale greenhouse shrimp culture is characterised by high salinity and high nitrogen levels. Currently, research on small-scale greenhouse ponds for *P. vannamei* has mostly focused on water quality control and healthy culture practices [18]. What factors significantly affect shrimp production in this farming mode? This study hypothesised that environmental bacterial communities and water quality factors affect shrimp production. Therefore, this study selected four representative shrimp aquaculture ponds from a small greenhouse aquaculture base in southern China and investigated the changes in microbial communities, water quality factors, and pathogens in pond water and shrimp intestines throughout the aquaculture process. Multiple linear regression equations and other analyses were used to identify potential factors affecting the production of *P. vannamei*, to provide data to support the healthy culture of this species in the small greenhouse model.

## 2. Materials and Methods

### 2.1. Sample Collection and Processing

The experimental ponds were placed in a *P. vannamei* small-scale greenhouse culture base in Jiangmen City, Guangdong Province, China. The base covers a 2.0 × 10^6^ m^2^ tract of land and is an important small greenhouse culture base in southern China. Four small-scale greenhouse ponds with the same management conditions were selected and numbered 1, 2, 3, and 4. Pond 4 was harvested before the shrimp were infected with the pathogens. Each pond had an area of 400 m^2^ and a water depth of 0.7 m. The *P. vannamei* were stocked on 9 October 2023 at a density of 70,000 tails per mu and a size of 1 cm per tail. All ponds were cleared of silt, dried, and disinfected before being filled with water. The pond water sources in this area were a mixture of seawater, groundwater, and river water, represented by HS, JS, and DS, respectively. During the culture stage, shrimp feed was provided four times a day. Every 2–3 days, photosynthetic bacteria (20 L per drum), lactic acid bacteria (260 g per bottle), EM bacteria (1 L per bottle), glutamate alginate (10 L per drum), and *Bacillus* spp. (500 g per bag) were sprinkled to control water quality.

Water quality and shrimp samples were collected four times, from September, October, and December 2023 to January 2024. Based on the culture stage, September was considered the baseline environmental sample, whereas October, December, and January were samples from the pre-, mid-, and late culture stages, corresponding to 15, 58, and 87 days of culture, respectively. The four samples were distinguished as P, A, B, and C, and the pond water, shrimp, and shrimp intestines were represented as W, S, and I, respectively. Different sampling points were selected at the front, middle, and back of the small greenhouse pond. Water samples were obtained from a depth of 0.5 m using a water sampler, and the water samples from the same pond were mixed and then divided according to the testing requirements for different indicators. Ten shrimp samples were randomly selected from each pond. Shrimp samples were collected on-site to measure body length and weight. Water samples were collected on-site to determine salinity, pH, dissolved oxygen, and temperature in the ponds. Water quality indicators, pathogens, shrimp samples, and microbial community composition samples were stored at −20 °C. Microbial community composition samples were collected from pond water and shrimp intestines. For pathogen detection and microbial community composition analysis, shrimp hepatopancreas and intestine samples were collected separately. Water quality samples were tested within 7 days. Pathogen samples were tested within 2 days. Microbial community samples from pond water, filtered through a 0.2 µm filter (PC membrane), were sent for testing along with shrimp intestine samples.

### 2.2. Water Quality Factors Testing

Based on the salinity of the monitored water, different water quality testing standards and methods were selected to measure of total phosphorus (TP), total nitrogen (TN), phosphate (${PO}_{4}^{3-}$-P), ammonium nitrogen (${NH}_{4}^{+}$-N), nitrite (${NO}_{2}^{-}$-N), nitrate (${NO}_{3}^{-}$-N), suspended substances (SS), dissolved oxygen (DO), and chemical oxygen demand (COD), which are the key indicators.

### 2.3. Pathogen Detection

Samples of 10 mL of pond water and 0.1 g of mixed hepatopancreas from shrimp were collected as pathogen test samples. Pathogen detection was performed using fluorescent quantitative PCR kits for AHPND, EHP, WSSV, and DIV1 (Xiamen Zhihui Lianfeng Biotechnology Co., Ltd., Xiamen, China). AHPND, EHP, WSSV, and DIV1 levels were measured using fluorescent quantitative PCR.

### 2.4. Analysis of Bacterial Community Composition

Bacterial DNA was isolated from shrimp intestinal and pond water samples by Mingke Biotechnology Co., Ltd. (Hangzhou, China). PCR was conducted using universal primers targeting the V3-V4 hypervariable region of the 16S rRNA gene in the bacterial DNA. After product recovery and purification, a library was constructed, and the PE250 sequencing strategy was used for sequencing. The obtained sequence data were subjected to quality control, trimming, denoising, assembly, and chimaera removal to generate a feature sequence table. QIIME2 (vsesion qiime2-2019.4) software and Silva132 database were used to identify and compare amplicon sequence variants (ASVs). Raw sequence data generated in this study were deposited in the NCBI for the Biotechnology Information Short Read Archive database (accession number PRJNA1214515).

Alpha diversity metrics were used to assess species diversity within individual habitats. QIIME (version 1.9.1) was used to compute the alpha diversity indices of the samples.

The main indices included the sequencing depth index (goods-coverage), observed species index (observed species), community richness index (Chao1), and diversity index (Shannon-Wiener diversity index).

### 2.5. Shrimp Growth Indicators

The calculation formulas for shrimp growth parameters are as follows:

(1) $$Specific\;Growth\;Rate\;\left(SGR\right)=\frac{\ln W_{1}-\ln W_{0}}{t}\times 100\%$$

(2) $${{Feed\;Conversion\;Ratio\;\left(FCR\right)=W}_{f}/(W}_{1}-W_{0})\times 100\%$$

where (W_1_) is the weight of shrimp at the end of the culture stage. (W_0_) is the initial weight of the shrimp at the start of the culture stage. (t) is the number of days in the culture stage. (W_f_) is the total weight of the feed consumed.

At least 10 shrimp from each pond were used to determine the growth indicators of *P. vannamei.* Apart from those, each sample was tested in triplicate for each indicator.

### 2.6. Data Analysis

Data were statistically analysed using Excel 2010 (IBM SPSS Statistics 17) and Origin 2022. The threshold for statistical significance was set at *p* < 0.05.

## 3. Results

### 3.1. Shrimp Growth Indicators and Culture Production

**Figure 1:**
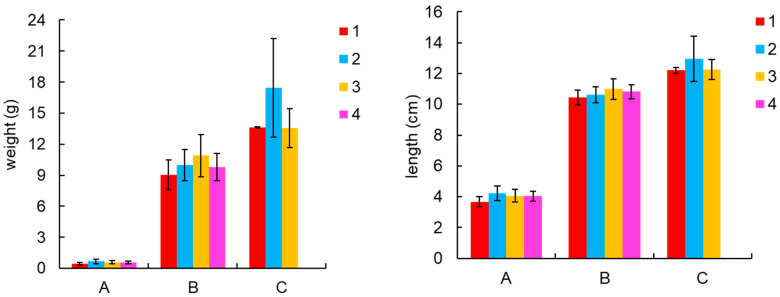
Growth performance of shrimp. A, B, and C represent the pre-, mid-, and late-culture stages, respectively, and 1, 2, 3, and 4 represent the four ponds.

The feed conversion ratio (FCR) for shrimp in each pond ranged from 1.34% to 1.36%, and the specific growth rate (SGR) was between 4.38% and 4.79%. No substantial variation was detected between the ponds (*p* > 0.05). Productions ranged from 2.12 to 2.70 kg·m^−2^. Pond 4 had a lower production than the other three ponds due to the early harvest caused by the disease (Table 1). Throughout the culture stage, the body length and weight of the shrimp continued to increase with the progression of the culture, especially during the mid-culture stage, when the shrimp grew more rapidly. The range of body length and weight of the shrimp was from 3.68 cm to 12.95 cm and from 0.43 g to 17.45 g, respectively (Figure 1).

### 3.2. Water Quality Indicators

In the source water, nitrite, nitrate, phosphate, TP, COD, and SS maintained relatively low levels throughout the culture stage, with values varied between 0.01 and 0.70 mg·L^−1^, 0.07 to 1.80 mg·L^−1^, 0.004 to 0.07 mg·L^−1^, 0.02 to 0.26 mg·L^−1^, 0.5 to 20 mg·L^−1^, and 0.004 to 0.23 g·L^−1^, respectively. The groundwater source had high concentrations of ammonia nitrogen and TN, ranging from 5.89 to 23.6 mg·L^−1^ and 13.8 to 27.3 mg·L^−1^, respectively. The pH of groundwater sources varied widely, ranging from 6.1 to 7.9. The salinity of the groundwater source was between 25 and 27. By the late stages of culture, the salinity of the seawater source had reached 26. The high salinity of the source water led to a slow increase in the salinity of the pond water in the greenhouse small-pond culture from 2 before culture to 26 during the late stages of culture.

**Figure 2:**
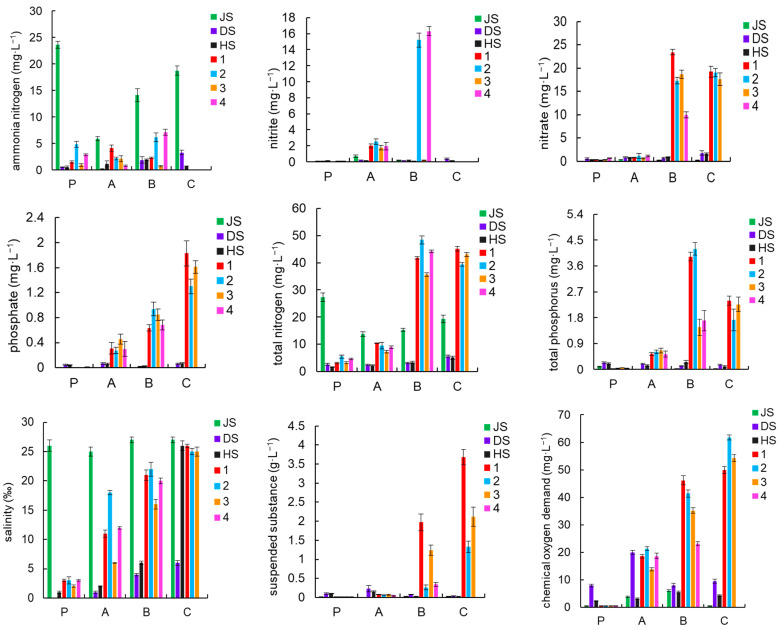
Characteristics of water quality indicator changes. Seawater, groundwater, and river water are represented by HS, JS, and DS, respectively; 1, 2, 3, and 4 represent the four ponds; and P, A, B, and C represent the baseline environment and pre-, mid-, and late-stage samples, respectively.

In the pond water, influenced by the high ammonia nitrogen concentration in the groundwater source, the ammonia nitrogen concentration was higher in the pre-and mid-stages of culture, with the highest concentration in Pond 4, reaching 7.13 mg·L^−1^, followed by Pond 2 at 6.15 mg·L^−1^. As nitrification progressed, the peak concentration of nitrite in the pond water occurred in the mid-stage of culture, with the highest concentration in Pond 4, reaching 16.3 mg·L^−1^, followed by Pond 2 at 15.2 mg·L^−1^. In the mid and late stages of culture, the nitrate level in the pond water ranged from 10.00 to 23.40 mg·L^−1^. TN levels were high during the mid and late stages, with concentrations ranging from 35.60 to 48.40 mg·L^−1^. Phosphate, COD, and SS showed a trend of gradual increase as the culture progressed, with concentrations in the late stages ranging from 1.30 to 1.83 mg·L^−1^, 50 to 61.7 mg·L^−1^, and 1.33 to 3.68 mg·L^−1^, respectively. The TP concentration reached high levels in the mid and late stages, ranging from 1.46 to 4.19 mg·L^−1^. The pH of the pond water was maintained between 7.1 and 7.8 (Figure 2).

### 3.3. Bacterial Community Composition in the Pond Water and Intestinal of Shrimp

#### 3.3.1. Bacterial Community Structure at the Phylum Level

**Figure 3:**
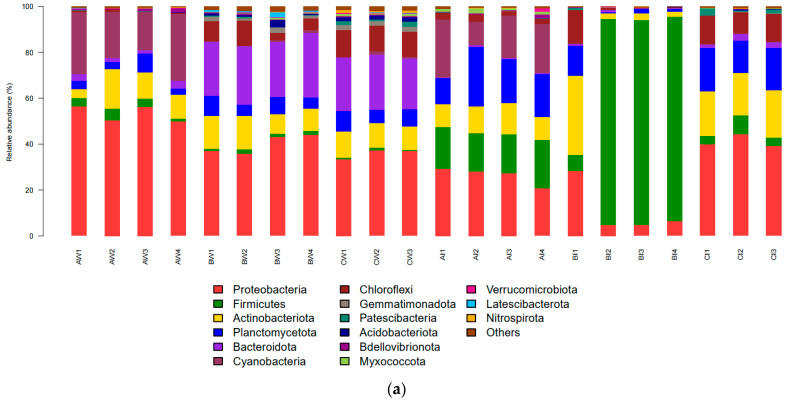

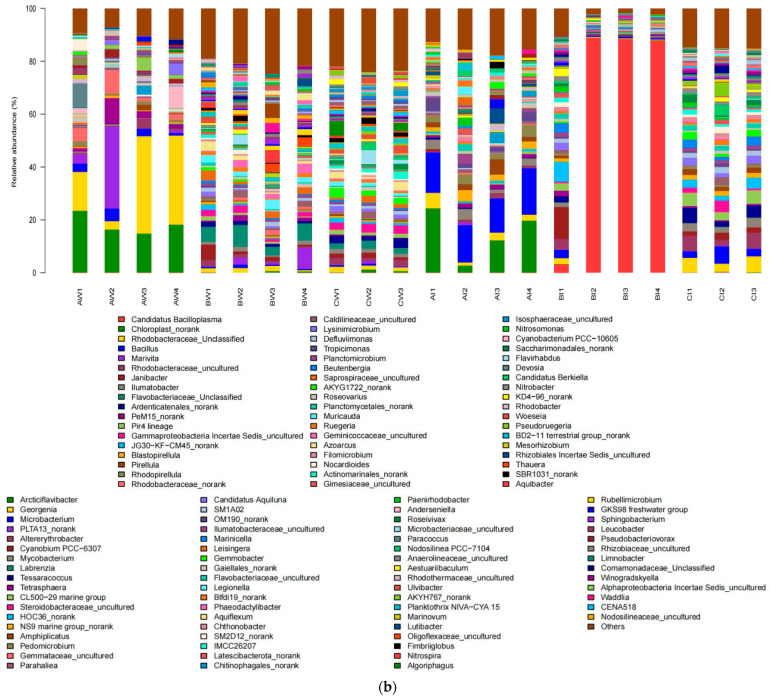

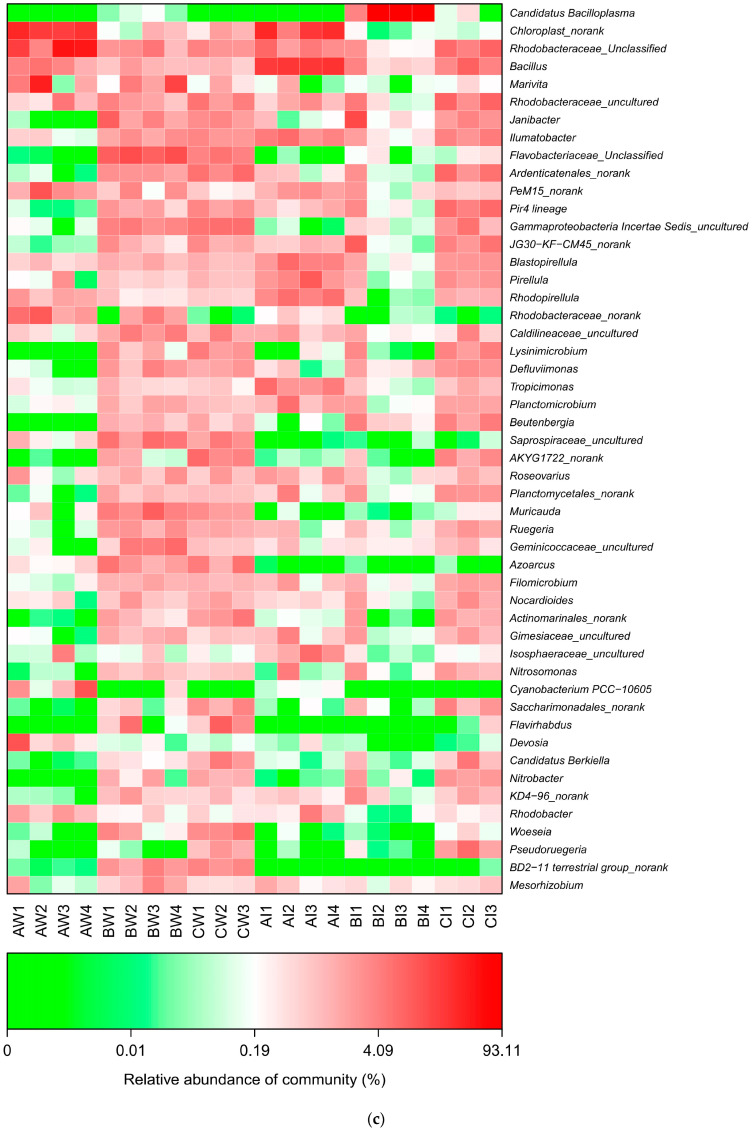

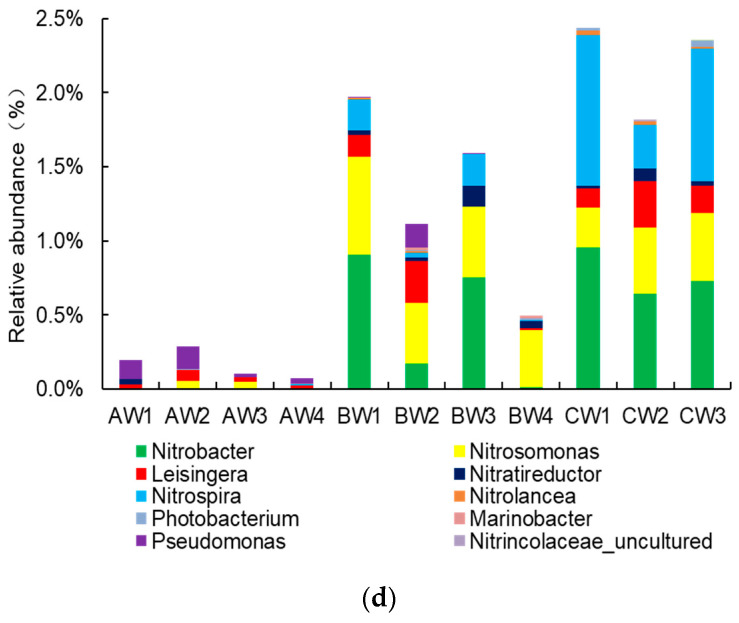
Bacterial community composition. (**a**) Phylum level; (**b**) genus level; (**c**) heatmap at genus level; (**d**) nitrifying bacteria at genus level. AW1, AW2, AW3, and AW4 represent pond water in four ponds during the pre-culture stage; BW1, BW2, BW3, and BW4 represent pond water in four ponds during the mid-culture stage; CW1, CW2, and CW3 represent pond water in three ponds during the late-culture stage; AI1, AI2, AI3, and AI4 represent shrimp intestines in four ponds during the pre-culture stage; BI1, BI2, BI3, and BI4 represent shrimp intestines in four ponds during the mid-culture stage; and CI1, CI2, and CI3 represent shrimp intestines in three ponds during the late-culture stage.

During the culture period, the dominant phyla in pond water and shrimp intestinal bacterial communities were *Proteobacteria* (43.72% and 24.83%), *Cyanobacteria* (8.83% and 6.93%), *Actinobacteriota* (11.09% and 13.29%), *Bacteroidota* (16.93% and 0.99%), *Planctomycetota* (6.02% and 13.02%), *Firmicutes* (2.01% and 33.06%), and *Chloroflexi* (5.85% and 5.59%, respectively) (Figure 3a).

During the preculture stage, the relative abundances of the dominant phyla in the pond water, from highest to lowest, were *Proteobacteria* (53.25%), *Cyanobacteria* (23.28%), *Actinobacteriota* (10.70%), *Planctomycetota* (4.45%), and *Firmicutes* (3.40%). In shrimp intestines, the order was *Proteobacteria* (26.28%), *Cyanobacteria* (18.89%), *Planctomycetota* (18.77%), *Firmicutes* (18.32%), and *Actinobacteriota* (11.32%).

During the mid-culture stage, the dominant phyla in the pond water, in descending order of abundance, were *Proteobacteria* (40.03%), *Bacteroidota* (25.39%), *Actinobacteriota* (11.76%), *Chloroflexi* (7.07%), and *Planctomycetota* (6.47%). In shrimp intestines, the orders were *Firmicutes* (68.74%), *Proteobacteria* (11.10%), *Actinobacteriota* (10.60%), *Chloroflexi* (4.22%), and *Planctomycetota* (4.19%).

During the late culture stage, the dominant phyla in the pond water, from highest to lowest abundance, were *Proteobacteria* (35.93%), *Bacteroidota* (23.06%), *Chloroflexi* (11.32%), *Actinobacteriota* (10.72%), and *Planctomycetota* (7.52%). In the shrimp intestines, the orders were Proteobacteria (41.21%), *Actinobacteriota* (19.50%), *Planctomycetota* (17.11%), *Chloroflexi* (11.41%), and *Firmicutes* (5.12%).

#### 3.3.2. Bacterial Community Structure at the Genus Level

At the genus level, the dominant bacterial genera in pond water and the intestinal tract of shrimp were *Ardenticatenales_norank* (1.27% and 1.47%), *Bacillus* (1.46% and 6.94%), *Candidatus Bacilloplasma* (0.02% and 24.43%), *Chloroplast_norank* (6.92% and 5.44%), *Flavobacteriaceae_unclassified* (2.84% and 0.11%), *Ilumatobacter* (0.96% and 2.11%), *Janibacter* (1.25% and 1.84%), *Marivita* (4.42% and 0.20%), *PeM15_norank* (1.77% and 0.94%), *Rhodobacteraceae_unclassified* (8.99% and 2.70%), and *Rhodobacteraceae_uncultured* (1.50% and 2.05%, respectively) (Figure 3b,c).

During the preculture stage, the relative abundances of the dominant genera in the pond water, in descending order, were *Rhodobacteraceae_unclassified* (22.04%), *Chloroplast_norank* (18.24%), *Marivita* (8.94%), *PeM15_norank* (3.69%), and *Bacillus* (2.95%). In the shrimp intestinal tract, the dominant genera were *Bacillus* (14.96%), *Chloroplast_norank* (14.80%), *Rhodobacteraceae_unclassified* (3.04%), and *Ilumatobacter* (3.02%).

During the mid-culture stage, the dominant genera in the pond water were *Flavobacteriaceae_unclassified* (5.97%) and *Marivita* (2.89%). At this stage, except for Pond 1, the dominant genus in the intestinal tracts of shrimp from Ponds 2, 3, and 4 was *Candidatus Bacilloplasma*, reaching a high abundance of 87.83–88.86%.

During the late culture stage, the dominant genera in the pond water were *Gammaproteobacteria Incertae Sedis_uncultured* (3.20%), *Arcticiflavibacter* (3.06%), and *Ardenticatenales_norank* (2.82%). In the shrimp intestinal tract, the dominant genera were *Rhodobacteraceae_unclassified* (4.85%) and *Rhodobacteraceae_uncultured* (4.68%).

#### 3.3.3. Nitrifying Bacterial Community in Pond Water at the Genus Level

Referring to the research on the types of nitrifying bacteria in shrimp pond culture by Xu et al. [8], the nitrifying bacteria identified in the pond water of this study included *Nitrobacter* (0.38%), *Nitrosomonas* (0.29%), *Nitrospira* (0.24%), *Leisingera* (0.11%), *Pseudomonas* (0.05%), *Nitratireductor* (0.04%), *Photobacterium* (0.009%), *Nitrolancea* (0.008%), *Marinobacter* (0.003%), *Nitrincolaceae* (0.001%), and *Nitrospina* (0.001%) (Figure 3d). During the preculture stage, the relative abundances of the dominant nitrifying bacterial genera, from the highest to lowest, were *Pseudomonas* (0.09%), *Leisingera* (0.04%), and *Nitrosomonas* (0.03%). During the mid-culture stage, the dominant nitrifying bacterial genera were *Nitrosomonas* (0.48%), *Nitrobacter* (0.46%) and *Leisingera* (0.11%). During the later culture stages, the dominant nitrifying bacterial genera were *Nitrosomonas* (0.78%), *Nitrobacter* (0.39%), and *Leisingera* (0.21%).

#### 3.3.4. Microbial Differences

**Figure 4:**
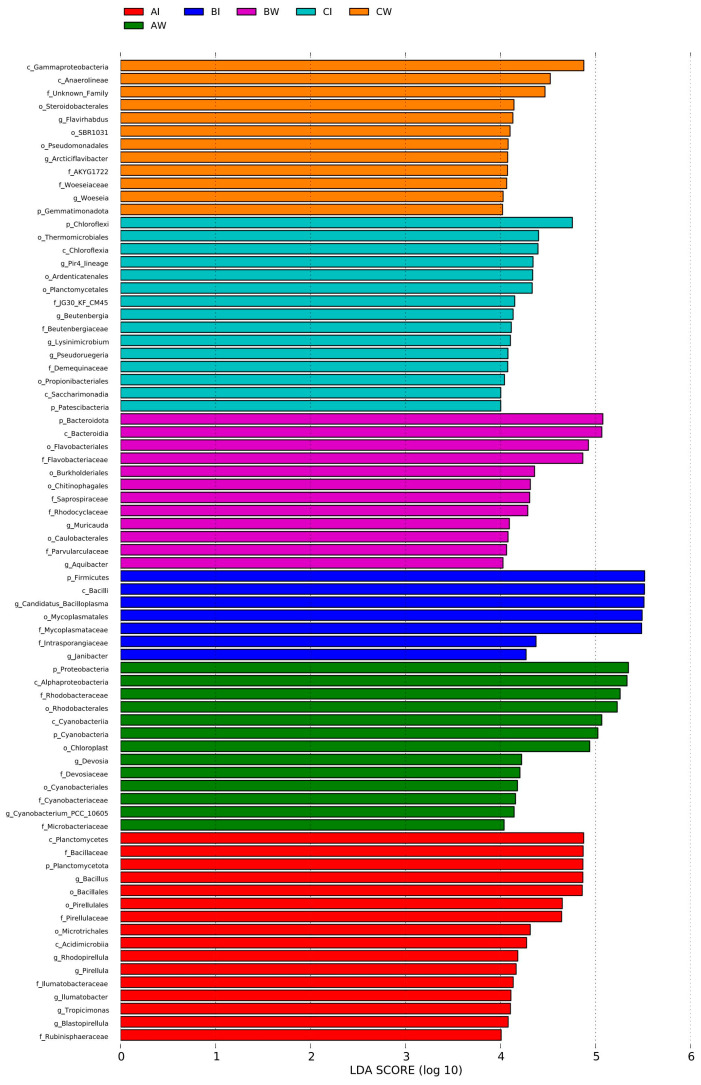
Microbial difference comparison. AW, pond water during the preculture stage; BW, pond water during the mid-culture stage; CW, pond water during the late-culture stage; AI, shrimp intestines during the pre-culture stage; BI, shrimp intestines during the mid-culture stage; CI, shrimp intestines during the late-culture stage.

Linear discriminant analysis (LDA) using LEfSe was conducted to identify the indicator species affecting the microbial communities in pond water and shrimp intestinal tracts across different culture stages (Figure 4). At an LDA score threshold of 4.5, 27 biomarkers were identified, including 7 at the phylum level, 7 at the class level, 6 at the order level, 5 at the family level, and 2 at the genus level, with statistical significance (*p* < 0.05).

At the phylum level, Proteobacteria, Cyanobacteria, Actinobacteriota, Bacteroidota, Planctomycetota, Firmicutes, and Chloroflexi were the dominant microorganisms in both pond water and the shrimp intestinal tract. At the class level, Alphaproteobacteria and Cyanobacteria indicated the pre-stage pond water, Planctomycetes indicated the pre-stage shrimp intestinal tract, Bacteroidota indicated the mid-stage pond water, and Firmicutes indicated the mid-stage shrimp intestinal tract; Gammaproteobacteria and Anaerolineae indicated late-stage pond water.

At the order level, Rhodobacterales and Chloroplast indicated the pre-stage pond water, Bacillales and Pirellulales indicated the pre-stage shrimp intestinal tract, Flavobacteriales indicated the mid-stage pond water, and Mycoplasmatales indicated the mid-stage shrimp intestinal tract.

At the family level, Rhodobacteraceae indicated the pre-stage pond water, Bacillaceae and Pirellulaceae indicated the pre-stage shrimp intestinal tract, Flavobacteriaceae indicated the mid-stage pond water, and Mycoplasmataceae indicated the mid-stage shrimp intestinal tract.

At the genus level, *Bacillus* indicated the pre-stage shrimp intestinal tract and *Candidatus Bacilloplasma* indicated the mid-stage shrimp intestinal tract.

#### 3.3.5. α Diversity Index Analysis

**Figure 5:**
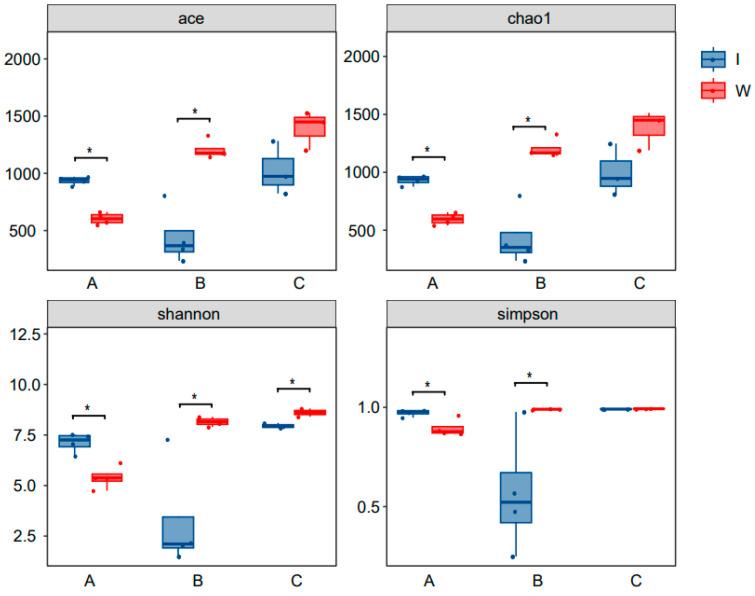
Alpha diversity index analysis. W and I represent pond water and shrimp intestines, and A, B, and C represent pre-, mid-, and late-stage samples, respectively; * represents *p* < 0.05.

Analysis of the α diversity indices revealed that the Shannon index showed significant differences in bacterial community diversity between the pond water and the shrimp intestinal tract at various stages. The Simpson index suggested notable differences in the diversity of the bacterial communities in pond water and shrimp intestinal tracts between the pre- and mid-culture stages. For the ACE and Chao1 indices, there were notable differences in the richness of the bacterial communities in the pond water and shrimp intestinal tract between the pre-and mid-culture stages (*p* < 0.05). The ACE, Chao1, and Shannon indices suggested that the diversity and richness of the bacterial communities in pond water gradually increased as the culture progressed, whereas the diversity and richness in the shrimp intestinal tract showed an initial decline followed by an increase, reaching their nadir in the mid-stage of culture (Figure 5).

#### 3.3.6. PCoA Analysis

**Figure 6:**
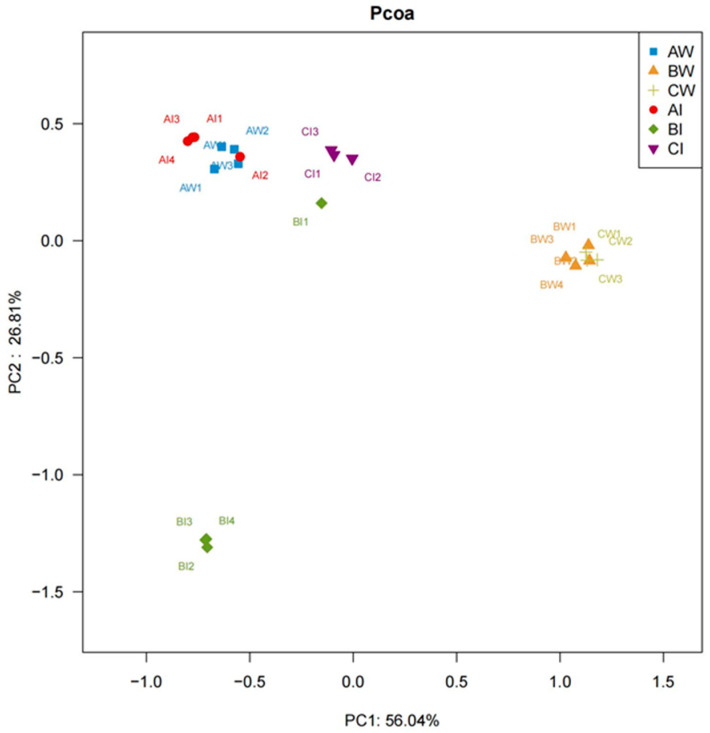
PCoA analysis. AW1, AW2, AW3, and AW4 represent pond water in four ponds during the pre-culture stage; BW1, BW2, BW3, and BW4 represent pond water in four ponds during the mid-culture stage; CW1, CW2, and CW3 represent pond water in three ponds during the late-culture stage; AI1, AI2, AI3, and AI4 represent shrimp intestines in four ponds during the pre-culture stage; BI1, BI2, BI3, and BI4 represent shrimp intestines in four ponds during the mid-culture stage; and CI1, CI2, and CI3 represent shrimp intestines in three ponds during the late-culture stage.

PCoA analysis was conducted on the microorganisms in the pond water and shrimp intestinal tract at different stages of the shrimp culture. The first principal coordinate axis contributed 56.04% of the sample variation, and the second principal coordinate axis contributed 26.81% of the sample variation. Microbial communities in the shrimp intestinal tract at different stages clustered separately, showing significant differences in the microbial composition. In the pre-stage of culture, the microbial partitions in the pond water and shrimp intestinal tracts were similar, with small differences in the microbial composition. In the middle and late stages of culture, the microbial partitions in the pond water were similar, indicating higher similarity in community composition (Figure 6).

### 3.4. Pathogen Quantification

During the culture process, pathogen DIV1 was not detected in the source or pond water. In the pre and late stages of culture, the levels of AHPND, EHP, and WSSV in the pond water and shrimps were below the detection limit. At the mid-stage of culture, the levels of AHPND, EHP, and WSSV in Ponds 1, 2, and 3 were below the detection limits. In Pond 4, the levels of pathogenic AHPND and EHP were above the detection limit, whereas those of WSSV were below the detection limit. The counts of AHPND and EHP in the pond water of Pond 4 were 78.93 and 1.09 × 10^3^ copies·mL^−1^, respectively, and the quantities in the shrimp were 2.18 × 10^3^ and 7.88 × 10^4^ copies·mg^−1^, respectively. The EHP content was higher than AHPND, and the pathogenic load in shrimp was higher than that in water (*p* > 0.05) (Table 2).

### 3.5. The Relationship Between Nitrifying Bacterial Communities and Water Quality Factors

**Figure 7:**
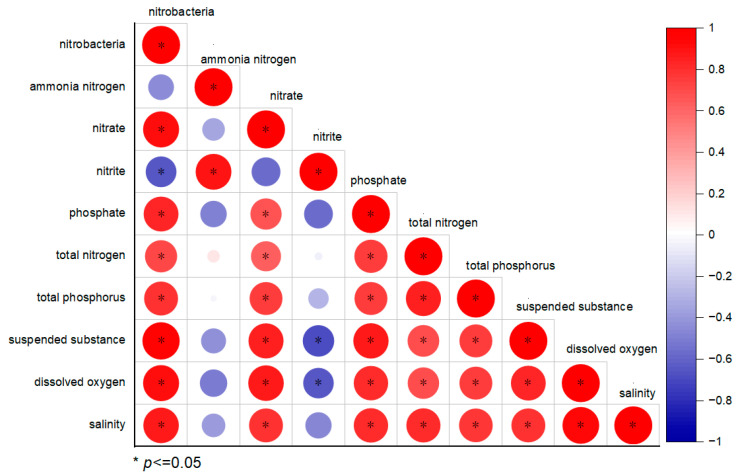
Relationship between nitrifying bacteria and water quality factors; * represents *p* < 0.05.

Spearman’s correlation analysis revealed a significant negative correlation between the abundance of nitrifying bacterial communities and nitrite concentration (*p* < 0.05), and a notable negative association with ammonia–nitrogen levels. In addition, there was a significant positive relationship between the abundance of nitrifying bacteria and TN, nitrate–nitrogen, suspended substances, dissolved oxygen, and salinity (*p* < 0.05) (Figure 7).

### 3.6. The Relationship Between Bacterial Communities, Pathogens, Water Quality Factors, and Shrimp Production

Using shrimp production as the dependent variable and the logarithmic values of the top 10 dominant bacterial genera, pathogens, and water quality factors as the independent variables, we analysed the impact of dominant bacterial communities, pathogens, and water quality factors on shrimp production. Through multiple stepwise linear regression analysis, the following regression equation was used:

(3) $$LgY=0.036X_{1}+0.029X_{2}-1.4\times 10^{-6}X_{3}+3.146$$

where X_1_, X_2_, and X_3_ represent the relative abundances of *Ardenticatenales_norank* and *Marivita* and the counts of EHP in pond water, respectively (Table 3).

## 4. Discussion

### 4.1. The Impact of the Pond Water Environment on Shrimp Culture

The quality of the aquatic water environment is crucial for the healthy growth of shrimp. Among the water quality indicators, ammonia nitrogen and nitrite are indicators of a good water environment [19]. High levels of ammonia, nitrogen, and nitrite can pose toxicity risks to shrimp; prolonged exposure to high concentrations can induce oxidative stress, impair the immune capacity of shrimp, and promote sublethal effects, leading to shrimp mortality [20,21]. In the small greenhouse culture model, the control of ammonia nitrogen and nitrite concentrations is crucial for the success of the culture [18]. Xu et al. [22] studied the indoor and outdoor culture processes of *P. vannamei* with biofloc regulation and found that nitrite concentrations initially increased and then decreased during the culture stage, peaking at the mid-culture stage. Xu et al. [23] found that nitrite reached its highest value during the mid-culture stage and gradually decreased to a stable level during the late-culture stage, whereas TN gradually increased during the culture stage, which is consistent with the trends of nitrite and TN observed in this study. In this study, the highest levels of ammonia nitrogen and nitrite in the pond water were recorded at 7.13 mg·L^−1^ and 16.3 mg·L^−1^, respectively, peaking during the mid-culture stage, followed by a decrease, after which ammonia nitrogen and nitrite were transformed into nitrate.

Studies have shown that increased salinity can reduce the sensitivity of *P. vannamei* to ammonia nitrogen and nitrite, leading to an increased tolerance [24]. For juvenile *P. vannamei*, the safe quality concentration of ammonia nitrogen is 2.83 mg·L^−1^ at low-salinity conditions [25]; the safe quality concentration of nitrite is 0.53 mg·L^−1^ [26]; at a salinity of 15~25, the safe quality concentration of ammonia nitrogen is 2.44~3.55 mg·L^−1^, and for nitrite, it is 6.1~15.2 mg·L^−1^; research indicates that the lethal dose of nitrite for juvenile *P. vannamei* is inversely proportional to the exposure amount and time, and directly proportional to the salinity [27,28]. This indicates that as time progressed and salinity increased, the tolerance of shrimp to ammonia nitrogen and nitrate during growth gradually increased. The study found that when the ammonia nitrogen level exceeds 5.0 mg·L^−1^ [29], the risk of stress in shrimp increased sharply. In this study, the ammonia nitrogen concentration in groundwater was 5.89~23.6 mg·L^−1^, and pond water comprised groundwater, seawater, and river water, so the pond water is inevitably influenced by the water source. Therefore, water sources should be used in aquaculture to prevent adverse effects. In addition, in the pre-stage of culture, the salinity range was 6–18, with the highest concentrations of ammonia nitrogen and nitrite at 4.09 and 2.53 mg·L^−1^, respectively, which are within the safe level for shrimp growth; in the mid-stage of culture, the salinity range was 16–22, with Ponds 2 and 4 having higher concentrations of ammonia nitrogen and nitrite, at 6.15 and 7.13 mg·L^−1^ for ammonia nitrogen, and 15.2 and 16.3 mg·L^−1^ for nitrite, which may have inhibited shrimp activity. However, since the culture was in the mid-stage, the salinity was higher, and the tolerance of shrimp to ammonia nitrogen and nitrite increased. Furthermore, as evidenced by shrimp body length and weight, the growth performance was best during the mid-stage of culture, with the most rapid growth (*p* < 0.05). Therefore, even in some ponds where the ammonia nitrogen and nitrite levels were slightly higher than the safe range, shrimp could still grow well. In the late stage of culture, the salinity range was 25–26, with the highest concentrations of ammonia nitrogen and nitrite at 0.02 and 0.009 mg·L^−1^, respectively, which are much lower than the safe range for shrimp growth. Although the concentrations of harmful nitrogenous compounds, such as ammonia nitrogen and nitrite, were higher during the mid-stage of culture in this study, they were all within the controllable limits, and shrimp growth was not affected.

### 4.2. The Impact of Microorganisms on Shrimp Culture

Microorganisms are crucial elements of aquaculture systems, greatly contributing to the stability of the aquatic environment and well-being of aquatic species [30,31].

Bacterial communities, as major components of microorganisms, have positive effects in monitoring pond environments and shrimp growth. At the phylum level, studies by Fang [18], Zhao [32], Zhan [33], Le [34], and others have found that Proteobacteria is the predominant bacterial phylum in pond water and shrimp intestines during shrimp aquaculture, like in the findings of this study. Previous studies have indicated that Proteobacteria contribute significantly to microbial community interactions and nutrient cycling in shrimp ponds [35]. In this study, the relative abundance of Proteobacteria in shrimp intestines ranged from 11.10% to 41.21%, with the lowest abundance observed in the mid-culture stage. According to the α diversity index analysis, the diversity of the bacterial community in the shrimp intestines was poor at this stage and highest at the late stage of culture, which may be related to the decrease in Proteobacteria. At the genus and family levels, the predominant bacteria in the pond water were *Marivita*, Rhodobacteraceae, and Flavobacteriaceae, whereas in the shrimp intestines, *Bacillus*, *Candidatus Bacilloplasma*, and Rhodobacteraceae were the dominant bacteria. *Marivita* is a denitrifying bacterium that participates in the cycling of carbon sources, reducing the accumulation of carbon sources and other substances in ponds [23,36]. In this study, the concentration of nitrate was higher in the mid and late stages of culture, whereas the abundance of this bacterium was lower during these stages, with a relative abundance of 0.43–2.89%, suggesting that it may have played a role in nitrogen reduction. Flavobacteriaceae is a group of bacteria that can use different types of carbon sources and produce a variety of antibiotics and other bioactive molecules [37]. Flavobacteriaceae was higher in the mid and late stages of culture, reaching a maximum of 8.20% As the culture progressed, substances in the water body accumulated, suggesting that this type of bacteria could use organic matter in the water to produce bioactive molecules. Rhodobacteraceae contributes to the ecological maintenance of animal health and water quality in biofloc-based aquaculture systems and has a high relative abundance in the intestines of healthy shrimp, making it a key indicator of shrimp health [9,38]. In this study, the regular sprinkling of photosynthetic and EM bacteria by farmers may be related to the higher relative abundance of these bacteria in the water, with Rhodobacteraceae having a higher relative abundance in the pre and late stages of culture, reaching up to 23.38% and a minimum of 3.77% and 2.66% in the mid-stage, respectively. The levels of nitrogen and phosphorus nutrients were higher in the mid stage, followed by the late stage, suggesting that these bacteria may have contributed to the reduction in N and P in the water quality. In shrimp intestines, the diversity of bacterial communities was higher in Pond 1, and the relative abundance of this bacterium was highest in Pond 1, reaching 11.31%, suggesting that it may contribute to the healthy growth of shrimp. The literature reports that *Bacillus* is a probiotic that can improve digestion and absorption, promote growth, and enhance immunity [39]. In this study, *Bacillus* was mainly present in the shrimp intestines and was highest in the pre-stage of culture, reaching up to 17.65%, whereas the shrimp grew most rapidly from the pre-to mid-stage, suggesting that *Bacillus* may have promoted the growth of shrimp. *Candidatus Bacilloplasma* is a potential pathogen with a higher relative abundance in the intestines of sick shrimp than in healthy shrimp and may adversely affect shrimp culture [11,40]. The study found that *Candidatus Bacilloplasma* was mainly present in Ponds 2, 3, and 4 during the mid-stage of culture, with a relative abundance of 87.83–88.86%; it is speculated that Pond 4 had a higher detection of EHP and AHPND in the shrimp, coupled with unstable bacterial communities, leading to shrimp disease and death. Because of the regular sprinkling of beneficial bacteria, such as photosynthetic and EM bacteria, to regulate the pond environment during culture, the relative abundance of beneficial bacteria, such as Rhodobacteraceae, increased in Ponds 2 and 3 during the late stage, and the bacterial communities gradually stabilised, allowing the culture to proceed smoothly. Among the nitrifying bacterial communities, *Nitrobacter* and *Nitrosomonas* were the dominant genera, like in the findings of Wu et al. [41]. The literature reports that these bacterial groups are the dominant nitrifying bacteria in wastewater biological denitrification processes and play a significant role in the nitrogen cycle by converting ammonia nitrogen into nitrite and nitrate [42]. In this study, the relative abundances of *Nitrosomonas* and *Nitrobacter* were the highest in the late stage of culture and the lowest in the mid-stage, reaching maxima of 0.78% and 0.39%, respectively. The concentration of nitrite was highest in the mid-stage and lowest in the late stage, suggesting that *Nitrosomonas* and *Nitrobacter* may have played a role in reducing the harmful nitrogen species nitrite.

Besides bacterial communities, the role of pathogens in shrimp cultures cannot be overlooked. In the past five years, newly emerging and frequently occurring shrimp diseases have mainly included EHP, AHPND, WSSV, and DIV1, which have led to shrimp mortality and reduced aquaculture productions [43,44]. In this study, all pathogens except DIV1 were detected, with higher quantities of EHP and AHPND present in Pond 4. This pond was also treated prematurely because of shrimp mortality, leading to the speculation that higher quantities of EHP and AHPND may be one reason for the impaired growth of shrimp in this pond. Therefore, it is important to closely monitor changes in related microorganisms to create a healthy aquaculture environment for shrimp production.

### 4.3. The Impact of Microbial and Water Quality Factor Regulation on Shrimp Culture Ponds

The use of microorganisms to regulate the quality of aquaculture water is a common environmental control method in shrimp culture. Adding probiotics to shrimp as dietary supplements or water additives can improve intestinal health and enhance nutrient absorption and disease resistance [45]. Li et al. [46] found that the addition of lactic acid bacteria could improve disease resistance and promote the growth of shrimp, whereas Li et al. [47] discovered that the addition of *Bacillus subtilis* and other *Bacillus* species could purify the aquaculture water environment. In this study, the water quality was regulated by the application of photosynthetic, lactic acid, and EM bacteria. In the pre-stage of culture, the water established beneficial microbial communities, such as *Bacillus* and Rhodobacteraceae. As the culture progressed, a nitrifying bacterial community was established in the water with a gradual increase in ammonia and nitrite nitrogen. Studies have shown a significant positive correlation between nitrifying bacteria such as *Nitrosospira* and nitrate [48]. Xu et al. [8] identified *Nitrosomonas* as the predominant genus among the ammonia- and nitrite-oxidising bacteria. This study revealed a substantial inverse relationship between the relative prevalence of nitrifying bacteria and nitrite levels, coupled with a markedly positive association with nitrate levels (*p* < 0.05). The relative abundance of nitrifying bacteria increased from 0.16% in the pre-stage to 2.20% in the late stage, indicating that establishing a nitrifying bacterial community in water was beneficial for the smooth transformation of ammonia nitrogen and nitrite nitrogen.

The multiple linear regression equation revealed a highly significant negative correlation between the EHP and shrimp production, which aligns with the findings of Patil et al. [49]. The relative abundance of *Ardenticatenales_norank* and *Marivita* in pond water was found to have a highly positive association with shrimp production. Literature reports that *Ardenticatenales_norank* and *Marivita* participate in denitrification by removing harmful substances such as nitrogen and maintaining the stability of the pond water environment, which is beneficial for shrimp growth [18]. In this study, although the levels of harmful nitrogen substances, such as ammonia nitrogen and nitrite, were high, the stability of the bacterial community in water was relatively high during the culture process. It is speculated that *Ardenticatenales_norank* and nitrifying bacteria play a role in reducing harmful nitrogen and nitrite, whereas *Marivita* contributes to carbon fixation and participates in the transformation of nitrite and nitrate, maintaining the stability of the pond environment.

In summary, establishing an optimal bacterial phase with denitrifying bacteria such as *Marivita* and nitrifying bacteria such as *Nitrosomonas* and Rhodobacteraceae, as well as controlling the pathogen load with a focus on EHP and AHPND, plays a significant role in regulating the pond environment and increasing shrimp culture production.

## 5. Conclusions

In the small-scale greenhouse pond for *P. vannamei* culture in the South China region, the source water was characterised by high TN, ammonia nitrogen, and salinity, whereas the pond water was characterised by high salinity and TN. Within the bacterial community, *Marivita,* Rhodobacteraceae, Flavobacteriaceae, *Bacillus*, and *Candidatus Bacilloplasma* were the dominant bacteria, and *Nitrosomonas* and *Nitrobacter* were the dominant nitrifying bacteria. Various pathogens such as AHPND, EHP, and WSSV have been identified in pond water and shrimp. Results from multivariate linear regression indicated that the counts of EHP in pond water, as well as the relative abundance of *Ardenticatenales_norank* and *Marivita*, greatly impacted shrimp production. Therefore, establishing an optimal bacterial community with denitrifying bacteria such as *Marivita* and nitrifying bacteria, such as *Nitrosomonas* and Rhodobacteraceae, as well as controlling the pathogen load with a focus on EHP and AHPND, greatly regulates the pond environment and enhances the production of shrimp cultures.

## Figures and Tables

**Table 1:** Growth indicators and culture production of shrimp.

Items	1	2	3	4
Production(kg·m^−2^)	2.50	2.62	2.70	2.12
Specific Growth Rate (%)	1.34	1.36	1.35	1.36
Feed Conversion Ratio (%)	4.79	4.53	4.38	/

Note: Numbers 1, 2, 3, and 4 represent four ponds.

**Table 2:** The counts of pathogens.

Breeding Stage	No.	AHPND(W)	EHP(W)	WSSV(W)	DIV1(W)	AHPND(S)	EHP(S)	WSSV(S)	DIV1(S)
A	JS	/	nd	/	nd	-	-	nd	-
	HS	/	nd	/	nd	-	-	nd	-
	DS	nd	nd	nd	nd	-	-	nd	-
	1	/	nd	nd	nd	nd	nd	nd	nd
	2	/	nd	nd	nd	/	nd	nd	nd
	3	nd	nd	/	nd	/	nd	nd	nd
	4	nd	nd	/	nd	nd	nd	nd	nd
B	JS	/	/	nd	nd	-	-	nd	-
	HS	/	/	nd	nd	-	-	nd	-
	DS	/	/	nd	nd	-	-	nd	-
	1	/	/	nd	nd	nd	/	nd	nd
	2	nd	/	nd	nd	/	/	nd	nd
	3	nd	/	/	nd	/	/	nd	nd
	4	78.93	1.09 × 10^3^	/	nd	2.18 × 10^3^	7.88 × 10^4^	nd	nd
C	JS	nd	nd	nd	nd	-	-	nd	-
	HS	nd	nd	nd	nd	-	-	nd	-
	DS	nd	nd	nd	nd	-	-	nd	-
	1	nd	nd	nd	nd	nd	nd	nd	nd
	2	nd	nd	/	nd	nd	nd	nd	nd
	3	nd	nd	/	nd	nd	nd	nd	nd

“/” indicates below the detection limit (copies < 50), “-” indicates not tested, and “nd” indicates not detected; seawater, groundwater, and river water are represented by HS, JS, and DS, respectively; 1, 2, 3, and 4 represent four ponds; A, B, and C represent the pre-, mid-, and late-stage samples; pond water and shrimp are represented by W and S.

**Table 3:** Output results of the relationship between the bacterial community, pathogens, water quality factors, and shrimp production.

Model a		Unstandardised Coefficients		Standardised Coefficients	T	Sig.	Collinearity Statistics	
		B	Std. Error	Beta			Tolerance	VIF
	(Constant)	3.146	0.029		109.116	0.000		
	EHP-W	−1.4 × 10^−6^	0.006	−0.871	−6.145	0.000	0.768	1.302
	Ardenticatenales_norank-W	0.036	0.006	1.001	5.630	0.001	0.488	2.050
	Marivita-W	0.029	0.008	0.684	3.586	0.009	0.424	2.361
a. Dependent: production								

W represents pond water.

## Data Availability

The datasets generated and analysed during the current study are available from the corresponding author on reasonable request.
